# Supplementary material for: A mathematical model for zoonotic transmission of malaria in the Atlantic Forest: Exploring the effects of variations in vector abundance and acrodendrophily
Source: PLoS Negl Trop Dis. 2021 Feb 16;15(2):e0008736. doi: 10.1371/journal.pntd.0008736 (PMC7909691; doi:10.1371/journal.pntd.0008736)
Supplement: S1 Text. The parameters ζc and ζg — (PDF) [file pntd.0008736.s001.pdf]

S1 Text. The parameters ζ_c and ζ_g.

By definition,

$$N_P + \zeta_c = (B_c + N_P)\left(1 + \frac{1}{h}\frac{C_c + M\,F_{mc}}{B_c + N_P}\right)$$

and

$$N_H + \zeta_g = (B_g + N_H)\left(1 + \frac{1}{h}\frac{C_g + M\,F_{mg}}{B_g + N_H}\right).$$

These equations are based on the proposal by Laporta et al. [1], which considers the dilution effect caused by non-host vertebrate animals (B_c and B_g) and the effect of competition between vectors (M) and non-vectors (C_c and C_g) for a blood meal source that reacts defensively after receiving a certain number of bites (h).

Rearranging the first of the two above equations, we obtain

$$(B_c + N_P)\left(1 + \frac{1}{h}\frac{C_c + M\,F_{mc}}{B_c + N_P}\right) = \frac{(N_P + B_c)h + M\,F_{mc} + C_c}{h}$$

$$= N_P + B_c + \frac{M\,F_{mc}}{h} + \frac{C_c}{h}$$

$$= N_P + \frac{B_c h + M\,F_{mc} + C_c}{h}.$$

Therefore,

$$\zeta_c = \frac{B_c h + M\,F_{mc} + C_c}{h}$$

and, following the same logic,

$$\zeta_g = \frac{B_g h + M\,F_{mg} + C_g}{h}.$$

REFERENCES

1. Laporta GZ, Prado PIKL De, Kraenkel RA, Coutinho RM, Sallum MAM. Biodiversity Can Help Prevent Malaria Outbreaks in Tropical Forests. PLoS Negl Trop Dis. 2013;7: e2139. doi:10.1371/journal.pntd.0002139
