# Supplementary material for: A mathematical model for zoonotic transmission of malaria in the Atlantic Forest: Exploring the effects of variations in vector abundance and acrodendrophily
Source: PLoS Negl Trop Dis. 2021 Feb 16;15(2):e0008736. doi: 10.1371/journal.pntd.0008736 (PMC7909691; doi:10.1371/journal.pntd.0008736)
Supplement: S2 Text — (PDF) [file pntd.0008736.s002.pdf]

S2 Text. Estimation of the parameters related to the simian host.

Given the lack of studies in the literature about *Plasmodium* transmission parameters for NHPs in the Atlantic Forest, we sought to estimate these parameters using the following strategy:

1) The data obtained by Deane et al. [1,2] in a study conducted in the city of Joinville, Brazil, were used. According to the study, human malaria was endemic in the region; around 46 % of the howler monkeys sampled were infected by *Plasmodium*; and from the total mosquitoes collected, approximately 70% were *An. cruzii*, 42% of which were collected close to the ground. The authors therefore assumed that in this scenario zoonotic transmission of *Plasmodium* was occurring;

2) We assumed that the proportion of the howler monkey population infected at the time was in equilibrium. Based on this assumption, we used Markov Chain Monte Carlo sampling method with an adaptive Metropolis algorithm [3,4] to identify parameter values that best fitted the equilibrium value. The best set of parameters were obtained using 2,000 iterations and a 95% highest posterior density interval was calculated for each parameter. Based on human transmission parameters estimated by Chitnis et al. [5], we set the initial values as τ = 0.0035, T_{PM} = 0.24 and T_{MP} = 0.022. The lower and upper bounds were assumed be τ = 0.001 - 0.01, T_{PM} = 0.024 – 0.48 and T_{MP} = 0.01 – 0.1. Different sets of simian parameters were fitted to different values of N_H $(10N_P, 5N_P, N_P)$, T_{HM} (0.24 and 0.024) and γ (0.0035 and 0.0055). The values of the other parameters were fixed previously: b=0.5, μ=0.8, T_{MH}= 0.022, , h= 20, F_{mc}= 0.58, F_{mg}= 0.42, , $C_{th} = 20(N_P + N_H), M = 0.7C_{th}$, $C_c = (C_{th} - M)\frac{1}{2}$, $C_g = C_c$, B_c=0 and B_g=0 (S1 Table).

REFERENCES

1. Deane LM., Ferreira Neto JA, Okumura M, Ferreira MO. Malaria parasites of Brazilian monkeys. Rev Inst Med Trop Sao Paulo. 1969;11(2): 71-86.

2. Deane LM, Deane MP, Ferreira Neto JA, Barbosa de Almeida F. On the transmission of simian malaria in Brazil. Rev Inst Med Trop Sao Paulo. 1971;13: 311–319.

3. Soetaert K, Petzoldt T. Inverse Modelling, Sensitivity and Monte Carlo Analysis in R Using Package FME. J Stat Softw. 2010;33. doi:10.18637/jss.v033.i03

4. Haario H, Saksman E, Tamminen J. An adaptive Metropolis algorithm. Bernoulli. 2001;7(2): 223-242.

5. Chitnis N, Hyman JM, Cushing JM. Determining important parameters in the spread of malaria through the sensitivity analysis of a mathematical model. Bull Math Biol. 2008;70: 1272–1296. doi:10.1007/s11538-008-9299-0
