# Supplementary material for: A mathematical model for zoonotic transmission of malaria in the Atlantic Forest: Exploring the effects of variations in vector abundance and acrodendrophily
Source: PLoS Negl Trop Dis. 2021 Feb 16;15(2):e0008736. doi: 10.1371/journal.pntd.0008736 (PMC7909691; doi:10.1371/journal.pntd.0008736)
Supplement: S3 Text — (PDF) [file pntd.0008736.s003.pdf]

S3 Text. Obtaining the basic reproduction number.

To obtain the basic reproduction number, there must be a disease-free equilibrium point [1]. For the proposed model, disease-free equilibrium occurs when $I_P = 0$, $S_P = N_P$, $I_H = 0$, $S_H = N_H$, $I_M = 0$ and $S_M = M$.

The infection compartments of the model can be represented by the two subsystems

$$\mathcal{F} = \begin{bmatrix} \frac{bT_{MP}N_pI_MF_{mc}}{N_P+\zeta_c} \\ \frac{bT_{MH}N_HI_MF_{mg}}{N_H+\zeta_g} \\ \frac{bT_{PM}MI_PF_{mc}}{N_P+\zeta_c} + \frac{bT_{HM}MI_HF_{mg}}{N_H+\zeta_g} \end{bmatrix} \quad \text{and} \quad \mathcal{V} = \begin{bmatrix} \tau I_P \\ \gamma I_H \\ \mu I_M \end{bmatrix},$$

where $\mathcal{F}$ describes the rates at which new infections occur and $\mathcal{V}$ the rates at which changes in state occur (including recovery and mortality). The linearization of the subsystems around the disease-free equilibrium point is then given by the Jacobian matrices of $\mathcal{F}$ and $\mathcal{V}$:

$$F = \begin{bmatrix} 0 & 0 & \frac{bT_{MP}N_PF_{mc}}{N_P+\zeta_c} \\ 0 & 0 & \frac{bT_{MH}N_HF_{mg}}{N_H+\zeta_g} \\ \frac{bT_{PM}MF_{mc}}{N_P+\zeta_c} & \frac{bT_{HM}MF_{Mg}}{N_H+\zeta_g} & 0 \end{bmatrix} \quad \text{and} \quad V = \begin{bmatrix} \tau & 0 & 0 \\ 0 & \gamma & 0 \\ 0 & 0 & \mu \end{bmatrix}.$$

The next generation matrix (K) is equal to FV^{-1}, i.e., matrix F multiplied by the inverse of matrix V. Thus, using

$$V^{-1} = \begin{bmatrix} \frac{1}{\tau} & 0 & 0 \\ 0 & \frac{1}{\gamma} & 0 \\ 0 & 0 & \frac{1}{\mu} \end{bmatrix},$$

we get

$$K = FV^{-1} = \begin{bmatrix} 0 & 0 & \frac{bT_{MP}N_P F_{mc}}{\mu(N_{P+}\zeta_c)} \\ 0 & 0 & \frac{bT_{MH}N_H F_{mg}}{\mu(N_{H+}\zeta_g)} \\ \frac{bT_{PM}MF_{mc}}{\tau(N_{P+}\zeta_c)} & \frac{bT_{HM}MF_{mg}}{\gamma(N_{H+}\zeta_g)} & 0 \end{bmatrix} = \begin{bmatrix} 0 & 0 & R_{MP} \\ 0 & 0 & R_{MH} \\ R_{PM} & R_{HM} & 0 \end{bmatrix}.$$

Hence,

$$R_0 = \sqrt{R_{MP}R_{PM} + R_{MH}R_{HM}} = \sqrt{\frac{bT_{MP}N_P F_{mc}}{\mu(N_{P+}\zeta_c)} \frac{bT_{PM}MF_{mc}}{\tau(N_{P+}\zeta_c)} + \frac{bT_{MH}N_H F_{mg}}{\mu(N_{H+}\zeta_g)} \frac{bT_{HM}MF_{mg}}{\gamma(N_{H+}\zeta_g)}}.$$

The equation $R_0 = \sqrt{R_{MH}R_{HM}} = \sqrt{\frac{bT_{MH}N_H F_{mg}}{\mu(N_{H+}\zeta_g)} \frac{bT_{HM}MF_{mg}}{\gamma(N_{H+}\zeta_g)}}$ is equivalent to the equation for R_0 in Laporta et al. [2] when $F_{mg} = 1$ and $\zeta_g = \frac{Bh+MF_{mg}+C}{h}$, where $B = B_c + B_g$ and $C = C_c + C_g$.

REFERENCES

1. Diekmann O, Heesterbeek JAP, Roberts MG. The construction of next-generation matrices for compartmental epidemic models. J R Soc Interface. 2010;7: 873–885. doi:10.1098/rsif.2009.0386

2. Laporta GZ, Prado PIKL De, Kraenkel RA, Coutinho RM, Sallum MAM. Biodiversity Can Help Prevent Malaria Outbreaks in Tropical Forests. PLoS Negl Trop Dis. 2013;7: e2139. doi:10.1371/journal.pntd.0002139
