# Supplementary material for: A mathematical model for zoonotic transmission of malaria in the Atlantic Forest: Exploring the effects of variations in vector abundance and acrodendrophily
Source: PLoS Negl Trop Dis. 2021 Feb 16;15(2):e0008736. doi: 10.1371/journal.pntd.0008736 (PMC7909691; doi:10.1371/journal.pntd.0008736)
Supplement: S1 Table — Alternative values for each parameter were estimated for different sets of human parameters. (DOCX) [file pntd.0008736.s004.docx]

**S1 Table.** **Estimation of simian parameters and 95% credible interval (highest posterior density) obtained by Markov Chain Monte Carlo sampling method.** Alternative values for each parameter were estimated for different sets of human parameters.

| Human parameters | | | | Estimated simian parameters (HPD 95%) | | |
| --- | --- | --- | --- | --- | --- | --- |
| *N_H_* | *T_MH_* | *T_HM_* | γ | τ | *T_PM_* | *T_MP_* |
| *10N_P_* | 0.022 | 0.24 | 0.0035 | 0.0044 (0.0016-0.0055) | 0.428 (0.105-0.471) | 0.034 (0.025-0.063) |
| *5N_P_* |  |  |  | 0.0044 (0.0037-0.0057) | 0.342 (0.249-0.479) | 0.029 (0.022-0.064) |
| *N_P_* |  |  |  | 0.0038 (0.0032-0.0044) | 0.261 (0.241-0.331) | 0.026 (0.019-0.03) |
| *10N_P_* | 0.022 | 0.024 | 0.0035 | 0.0027 (0.0025-0.0039) | 0.38 (0.339-0.479) | 0.04 (0.035-0.045) |
| *5N_P_* |  |  |  | 0.0027 (0.0025-0.0055) | 0.346 (0.262-0.477) | 0.03 (0.025-0.048) |
| *N_P_* |  |  |  | 0.0028 (0.0024-0.0030) | 0.291 (0.234-0.396) | 0.026 (0.020-0.033) |
| *10N_P_* | 0.022 | 0.024 | 0.0055 | 0.0025 (0.0012-0.0056) | 0.348 (0.224-0.477) | 0.041 (0.032-0.079) |
| *5N_P_* |  |  |  | 0.0031 (0.0020-0.0038) | 0.306 (0.302-0.432) | 0.04 (0.02-0.042) |
| *N_P_* |  |  |  | 0.003 (0.0028-0.0056) | 0.308 (0.301-0.429) | 0.028 (0.022-0.04) |
