# Supplementary figures and images for: A mathematical model for zoonotic transmission of malaria in the Atlantic Forest: Exploring the effects of variations in vector abundance and acrodendrophily
Source: PLoS Negl Trop Dis. 2021 Feb 16;15(2):e0008736. doi: 10.1371/journal.pntd.0008736 (PMC7909691; doi:10.1371/journal.pntd.0008736)

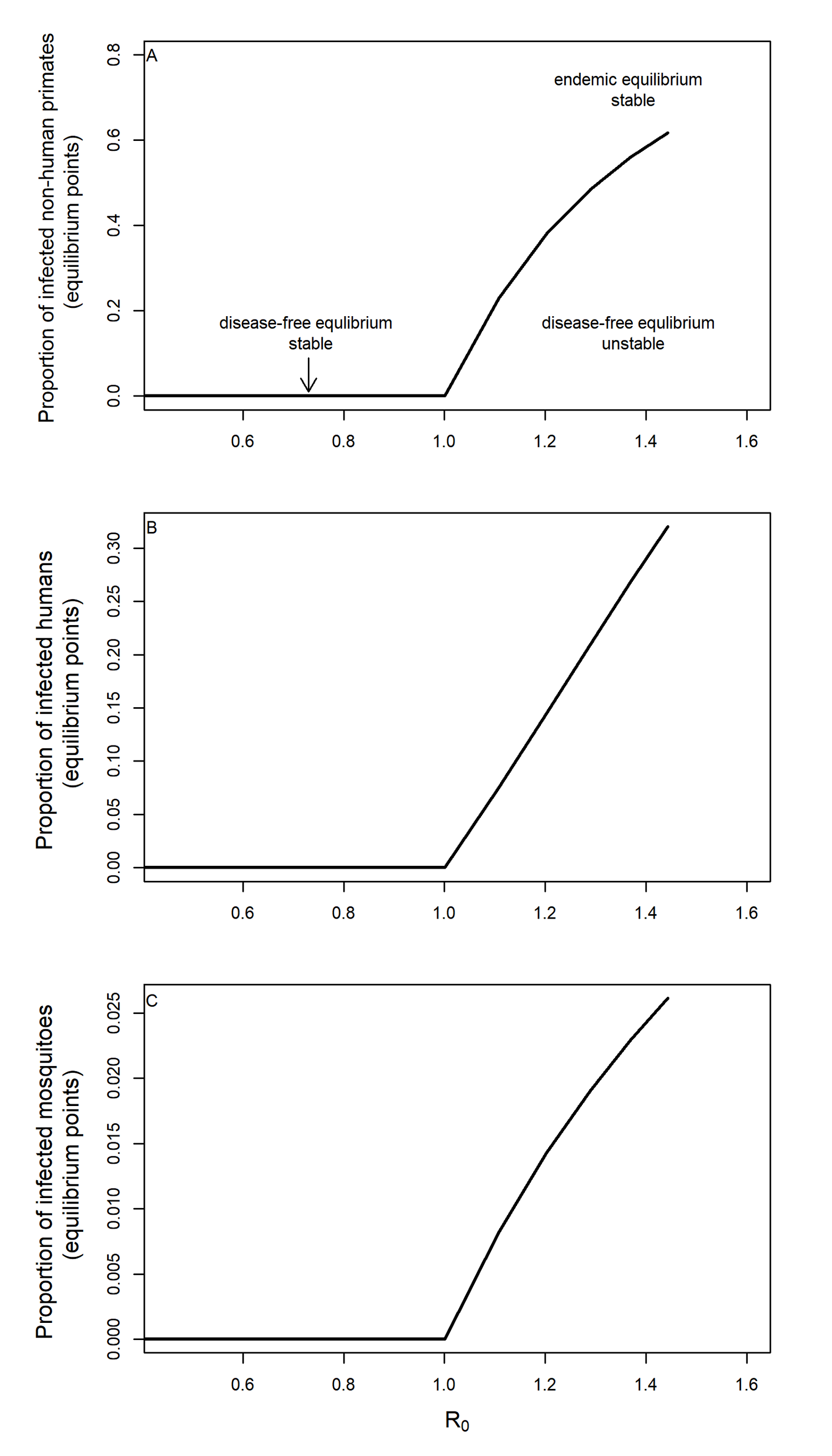

Supplement: S1 Fig — The R0 values were obtained by the variation of M (from 0.013 to 0.91Cth). The other parameters were set at: b = 0.5, μ = 0.8, TMH = 0.022, THM = 0.24, γ = 0.0035, h = 20, Fmc = 0.58, Fmg = 1−Fmc, NH = 300, NP = 30, Cth = 20(NP+NH), Cc=(Cth−M)12, Cg = Cc, Bc = 0, Bg = 0, τ = 0.0044, TMP = 0.034, and TPM = 0.428. (TIF) [file pntd.0008736.s005.tif]

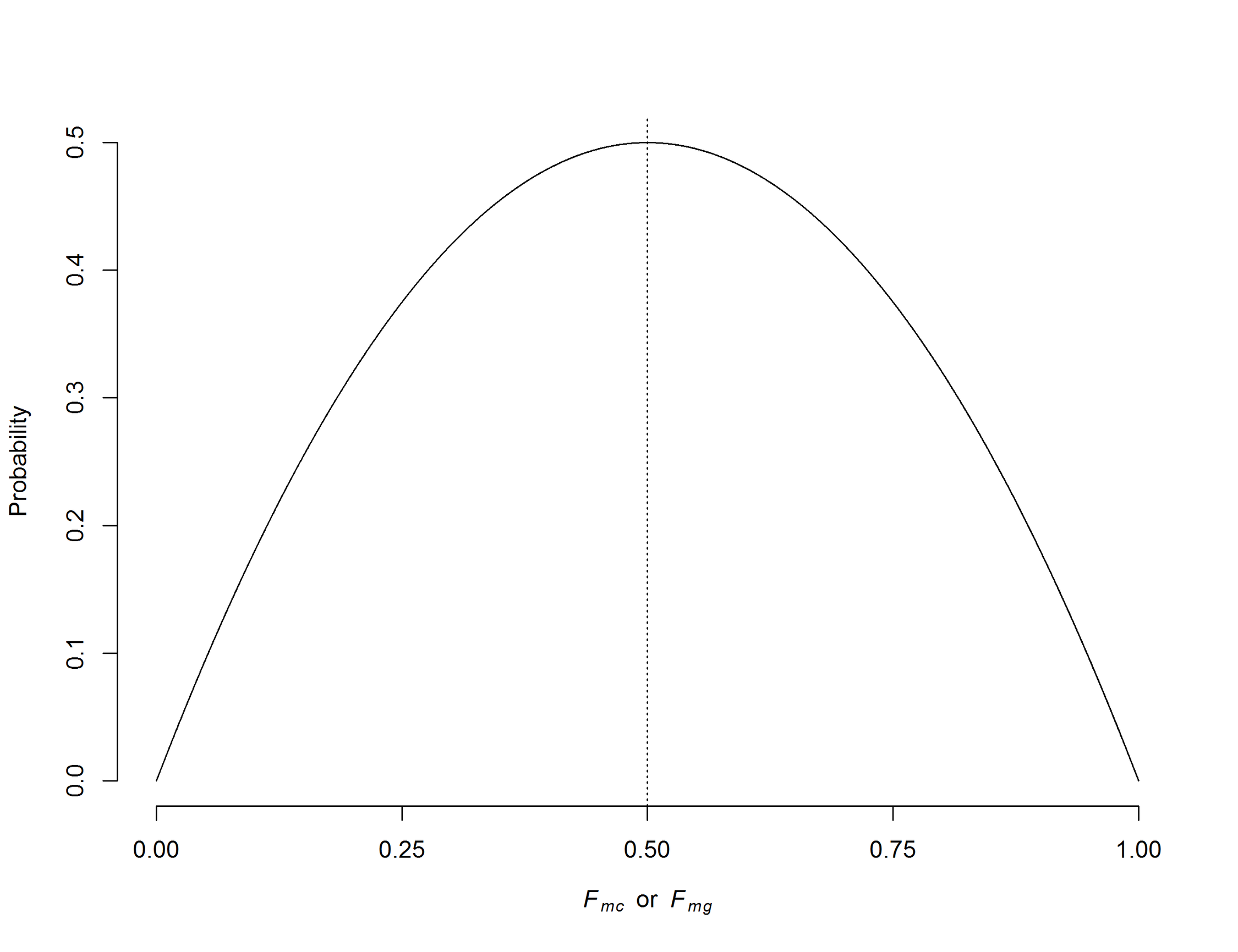

Supplement: S2 Fig — The vertical axis corresponds to the probability that the same mosquito bites first in the canopy and then at ground level (or vice versa), which equals 2FmcFmg. The dotted line represents the maximum probability, which occurs when Fmc = Fmg = 0.5. (TIF) [file pntd.0008736.s006.tif]

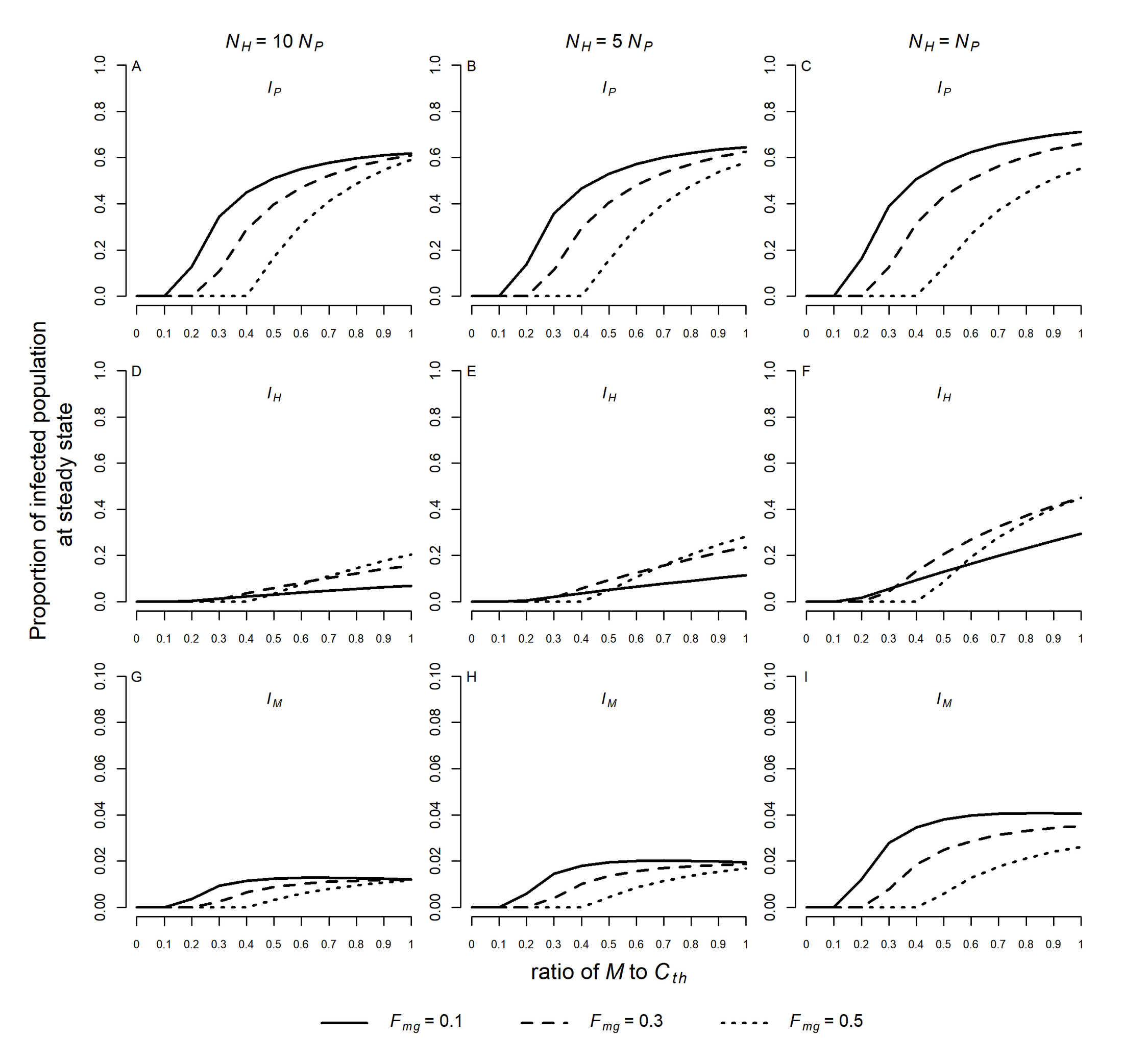

Supplement: S3 Fig — Proportions of infected individuals at steady state in the monkey (A, B, C), human (D, E, F), and mosquito (G, H, I) populations when THM = 0.024. Simulations were performed for a ratio of M to Cth varying from 0.01 to 0.99 and for Fmg = 0.1, 0.3 and 0.5. Three different scenarios were considered: NH = 10NP (A, D, G), NH = 5NP (B, E, H), and NH = NP (C, F, I). The following values were assumed to simian parameters: τ = 0.0027, TMP = 0.04, and TPM = 0.38 when NH = 10NP; τ = 0.0027, TMP = 0.03, and TPM = 0.346 when NH = 5NP; τ = 0.0028, TMP = 0.026, and TPM = 0.291 when NH = NP. The values of the other parameters used in the model were fixed: Cth = 20(NH + NP), Cc=(Cth−M)12, Cg = Cc, μ = 0.8, γ = 0.0035, TMH = 0.022, TMP = 0.044, b = 0.5, h = 20, Bc = 0 and Bg = 0. (TIF) [file pntd.0008736.s007.tif]

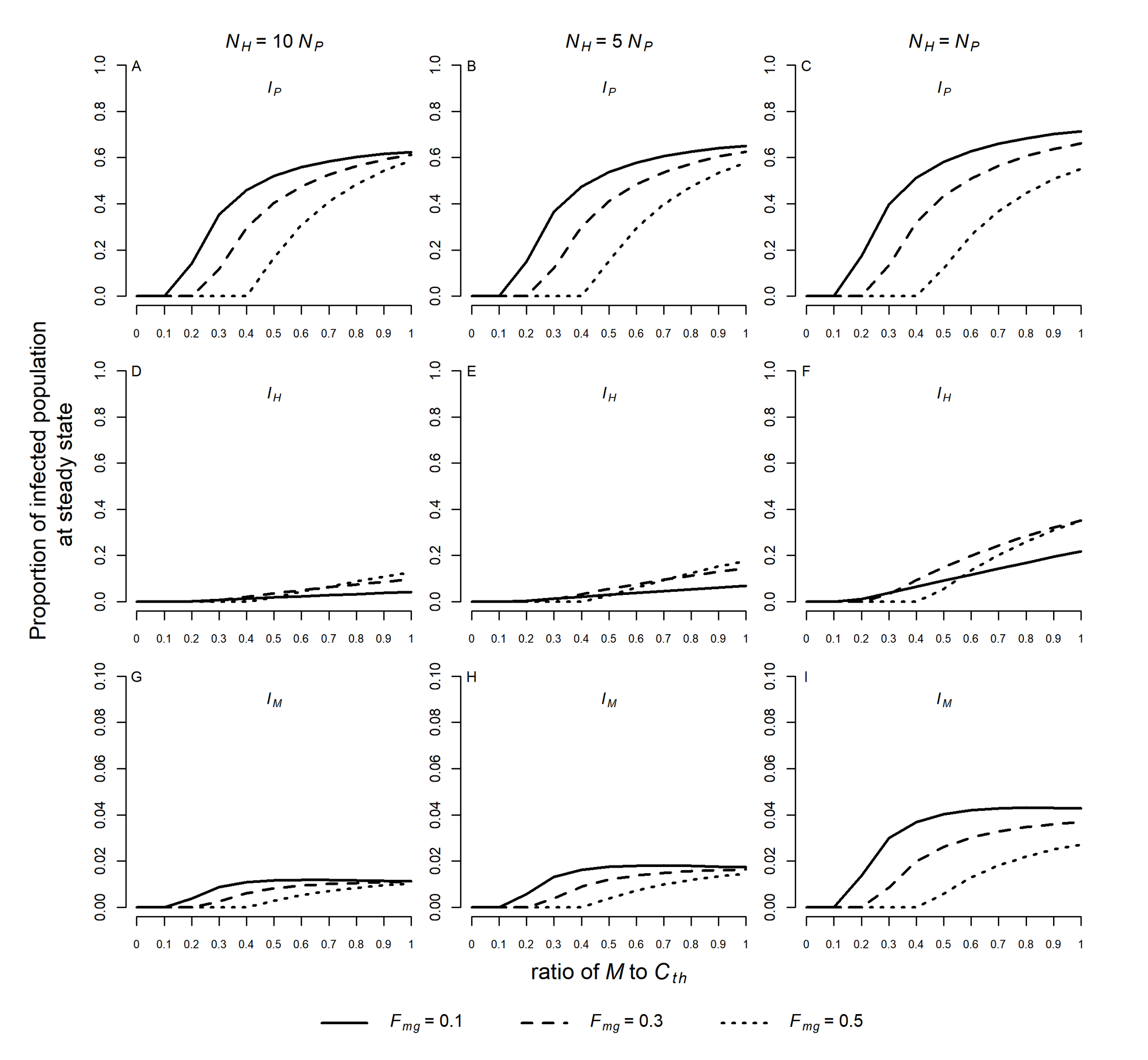

Supplement: S4 Fig — Proportions of infected individuals at steady state in the monkey (A, B, C), human (D, E, F), and mosquito (G, H, I) populations when THM = 0.024 and γ = 0.0055. Simulations were performed for a ratio of M to Cth varying from 0.01 to 0.99 and for Fmg = 0.1, 0.3 and 0.5. Three different scenarios were considered: NH = 10NP (A, D, G), NH = 5NP (B, E, H), and NH = NP (C, F, I). The following values were assumed to simian parameters: τ = 0.0025, TMP = 0.041, and TPM = 0.348 when NH = 10NP; τ = 0.0031, TMP = 0.04, and TPM = 0.306 when NH = 5NP; τ = 0.003, TMP = 0.028, and TPM = 0.308 when NH = NP. The values of the other parameters used in the model were fixed: Cth = 20(NH + NP), Cc=(Cth−M)12, Cg = Cc, μ = 0.8, TMH = 0.022, TMP = 0.044, b = 0.5, h = 20, Bc = 0 and Bg = 0. (TIF) [file pntd.0008736.s008.tif]

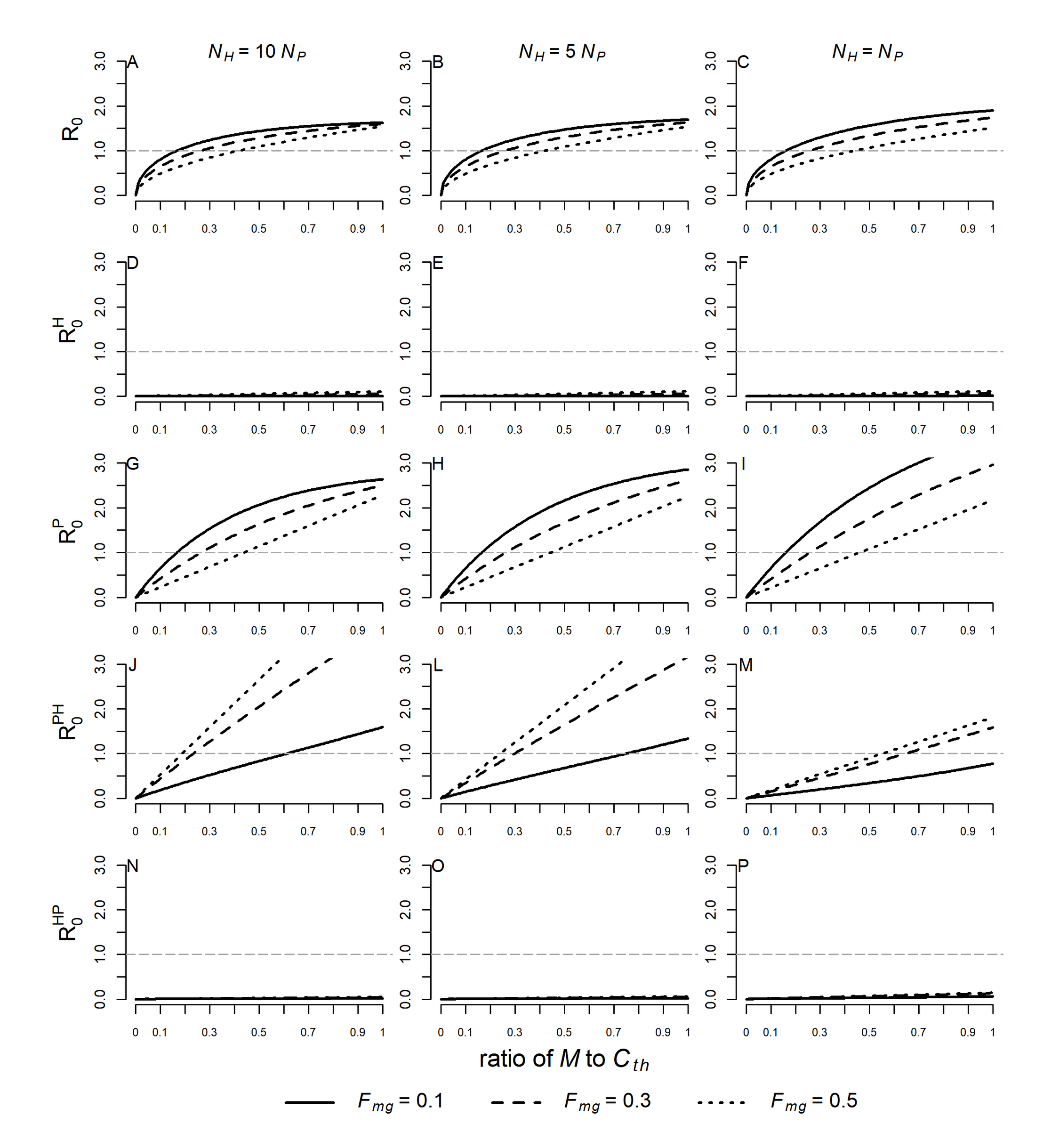

Supplement: S5 Fig — The dashed line represents the epidemic threshold, above which more than one new case will be generated in the susceptible population by an infected individual. Values were simulated for ratios of M to Cth ranging from 0.01 to 0.99 and for Fmg = 0.1, 0.3, and 0.5. Three different scenarios were considered: NH = 10NP (A, D, G, J, N), NH = 5NP (B, E, H, L, O) and NH = NP (C, F, I, M, P). The following values were assumed to simian parameters: τ = 0.0027, TMP = 0.04, and TPM = 0.38 when NH = 10NP; τ = 0.0027, TMP = 0.03, and TPM = 0.346 when NH = 5NP; τ = 0.0028, TMP = 0.026, and TPM = 0.291 when NH = NP. The values of the other parameters used in the model were fixed: Cth = 20(NH + NP), Cc=(Cth−M)12, Cg = Cc, μ = 0.8, γ = 0.0035, TMH = 0.022, TMP = 0.044, b = 0.5, h = 20, Bc = 0 and Bg = 0. (TIF) [file pntd.0008736.s009.tif]

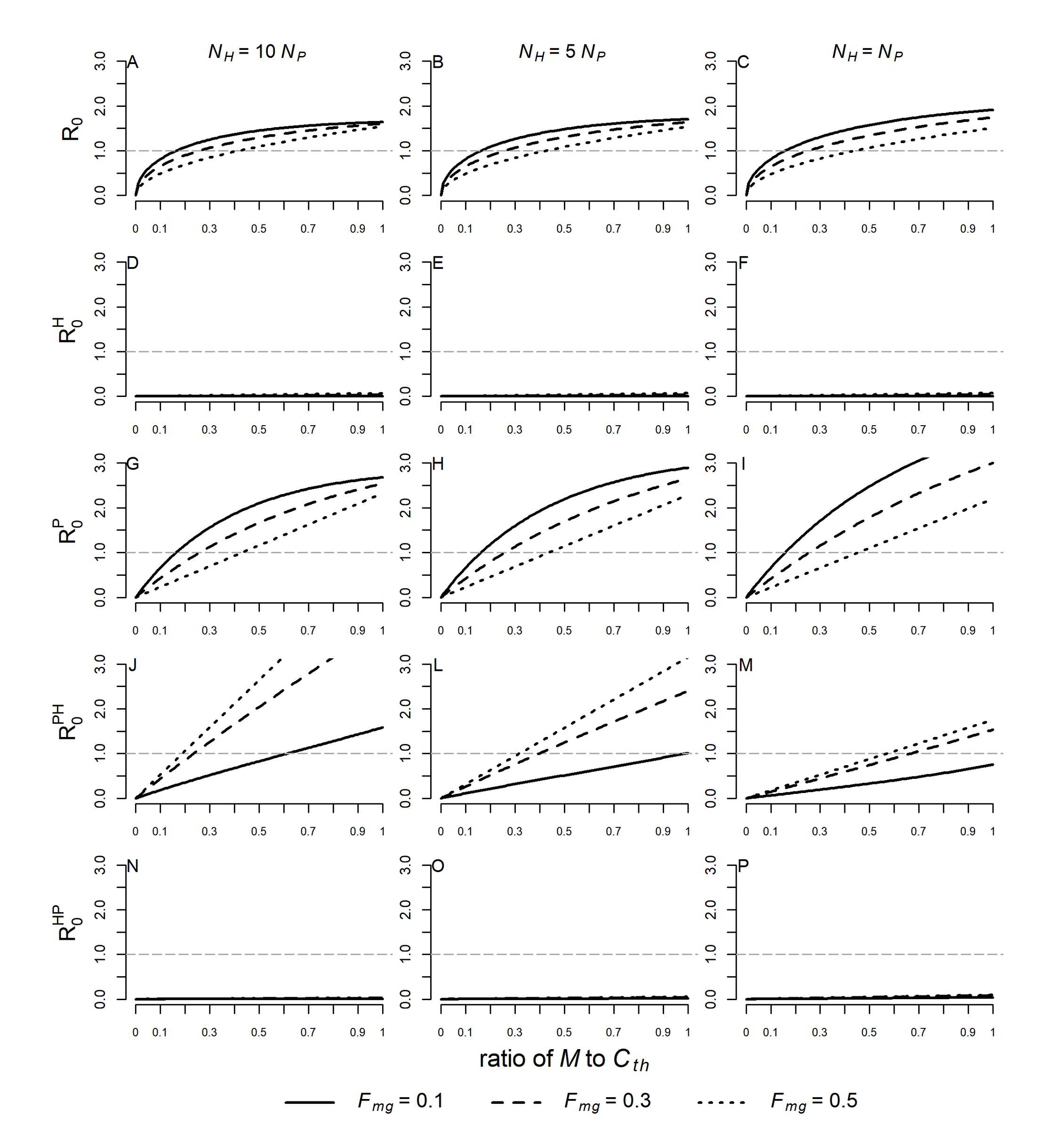

Supplement: S6 Fig — The dashed line represents the epidemic threshold, above which more than one new case will be generated in the susceptible population by an infected individual. Values were simulated for ratios of M to Cth ranging from 0.01 to 0.99 and for Fmg = 0.1, 0.3, and 0.5. Three different scenarios were considered: NH = 10NP (A, D, G, J, N), NH = 5NP (B, E, H, L, O) and NH = NP (C, F, I, M, P). The following values were assumed to simian parameters: τ = 0.0025, TMP = 0.041, and TPM = 0.348 when NH = 10NP; τ = 0.0031, TMP = 0.04, and TPM = 0.306 when NH = 5NP; τ = 0.003, TMP = 0.028, and TPM = 0.308 when NH = NP. The values of the other parameters used in the model were fixed: Cth = 20(NH + NP), Cc=(Cth−M)12, Cg = Cc, μ = 0.8, TMH = 0.022, TMP = 0.044, b = 0.5, h = 20, Bc = 0 and Bg = 0. (TIF) [file pntd.0008736.s010.tif]
